# FEM Modeling of the Relationship between the High-Temperature Hardness and High-Temperature, Quasi-Static Compression Experiment

**DOI:** 10.3390/ma11010034

**Published:** 2017-12-26

**Authors:** Tao Zhang, Feng Jiang, Lan Yan, Xipeng Xu

**Affiliations:** 1 Institute of Manufacturing Engineering, National Huaqiao University, Xiamen 361021, China; zhangtao.ime@hqu.edu.cn (T.Z.); xpxu@hqu.edu.cn (X.X.); 2 College of Mechanical Engineering and Automation, National Huaqiao University, Xiamen 361021, China; yanlan@hqu.edu.cn

**Keywords:** high temperature hardness, high temperature quasi-static compression experiment, FE simulation, thermal softening, stress distribution, strain distribution

## Abstract

The high-temperature hardness test has a wide range of applications, but lacks test standards. The purpose of this study is to develop a finite element method (FEM) model of the relationship between the high-temperature hardness and high-temperature, quasi-static compression experiment, which is a mature test technology with test standards. A high-temperature, quasi-static compression test and a high-temperature hardness test were carried out. The relationship between the high-temperature, quasi-static compression test results and the high-temperature hardness test results was built by the development of a high-temperature indentation finite element (FE) simulation. The simulated and experimental results of high-temperature hardness have been compared, verifying the accuracy of the high-temperature indentation FE simulation.The simulated results show that the high temperature hardness basically does not change with the change of load when the pile-up of material during indentation is ignored. The simulated and experimental results show that the decrease in hardness and thermal softening are consistent. The strain and stress of indentation were analyzed from the simulated contour. It was found that the strain increases with the increase of the test temperature, and the stress decreases with the increase of the test temperature.

## 1. Introduction

A hardness test is an accurate, simple, and non-destructive measurement of the mechanical properties of material. The hardness of a material has been extensively studied and involves mechanical [1], material [2,3] and other areas. The indentation hardness test is the primary method. In the test process, the size of the impression left after a hard indenter is pressed into the surface is measured to evaluate material hardness. The type of indenter mainly includes the Vickers indenter [2,4,5], Brinell indenter [6] and Knoop indenter [7]. The indentation hardness test method could also be used to acquire high-temperature hardness. The high-temperature hardness test method has been widely used on a variety of materials, such as metal [8,9,10], ceramics [11], and polymer material [12]. The object of the high-temperature hardness test may be the matrix [13] or coating [14]. Thus, the application areas of a high-temperature hardness test are wide, and it has been widely used to study the effect of material formulation [15], preparation process [12], phase transition [16] and other factors of the mechanical properties under high temperature conditions. However, there are still many technical problems for high-temperature hardness tests. Firstly, it is difficult to eliminate the effect of high temperature on tester stiffness. Secondly, it is a huge challenge to acquire the indentation size under high temperature conditions. Thirdly, high-temperature oxidation has a great impact on the test result and indenter life. The most serious problem is the lack of high-temperature hardness test standards, which constrain its development. Thus, it is necessary to study how to develop the relationship between high-temperature hardness and standard mechanical performance test results.

The relationship between hardness and other mechanical properties acquired by standard mechanical performance tests has been extensively studied. Some scholars have studied the relationship between hardness and other mechanical properties, such as elastic modulus [17,18,19,20], yield strength [21], and ultimate tensile streng [22]. The most widely accepted view is that there is a correlation between the hardness *H* and the yield strength *σ_s_*, which could be expressed as the following equation [21]:

$$H=C\sigma_{S}$$

where *C* is the constraint factor for the hardness test, and *C* approximates three for the Brinell, Vickers and Knoop hardness tests. This conclusion has been confirmed by many scholars [23].

The FEM could be used to study the material deformation process more deeply, and it is an effective means to research the indentation process [24,25] and to establish the relationship between the standard mechanical performance test and hardness. The relationship between hardness and strain [26], and the relationship between hardness and effective stress [27] have been developed by FEM. The hardness test and uniaxial compression test are also the measurement methods for resistance to plastic deformation; it is important to relate them together by FEM [28], and this has been successfully applied under room temperature conditions [26,29].

The purpose of this study is to build the FEM model of the relationship between the high-temperature hardness and the high-temperature, quasi-static compression experiment by the development of a high temperature indentation FE simulation. A high-temperature, quasi-static compression test and a high-temperature hardness test were carried out, and they were related by the development of high-temperature indentation FE simulation. The results of the high-temperature indentation simulation and high-temperature hardness experiment have been compared to verify the accuracy of the high-temperature indentation FE simulation. The strain and stress of indentation have also been analyzed.

## 2. Experiments

**Figure 1:**
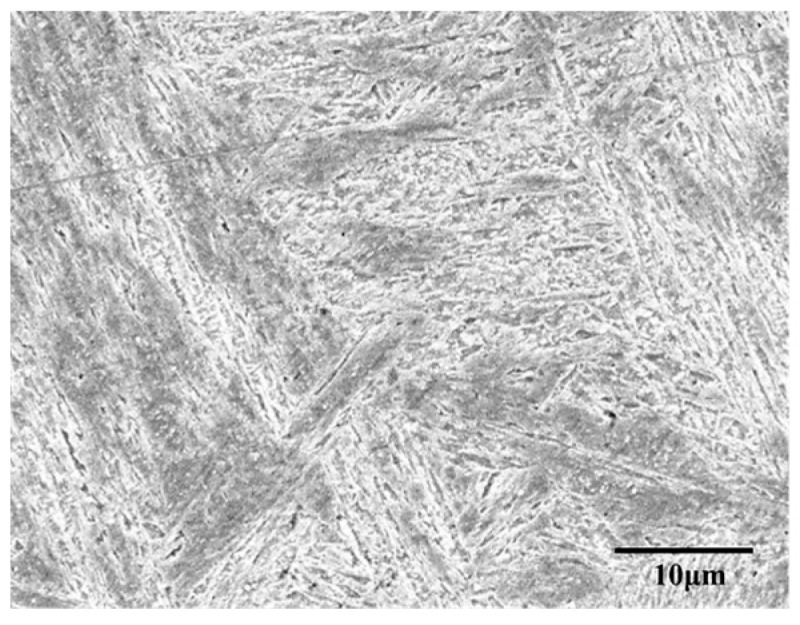
The microstructure of the sample material (SEM).

Fe-Cr-Ni stainless steel, which is a kind of austenitic stainless steel, has been commonly used for steam turbine blades, which are usually operated in an environment higher than 400 °C. The chemical composition of the sample material is shown in Table 1, and the microstructure of the sample material acquired by scanning electron microscope (SEM) is shown in Figure 1.

### 2.1. High-Temperature, Quasi-Static Compression Experiments

A series of high-temperature, quasi-static uniaxial compression tests were carried out in an Instron 8875 universal testing machine, and the design of experiment is shown in Table 2.

**Figure 2:**
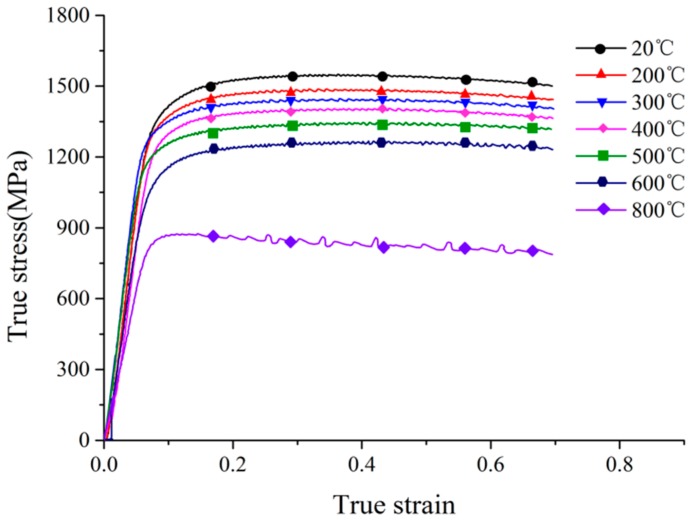
True-stress–true-strain curves at different test temperatures.

The engineering-strain–engineering-stress curves under different test temperatures were acquired, then they were converted to true-stress–true-strain curves, which are shown in Figure 2.

### 2.2. High-Temperature Hardness Experiments

**Figure 3:**
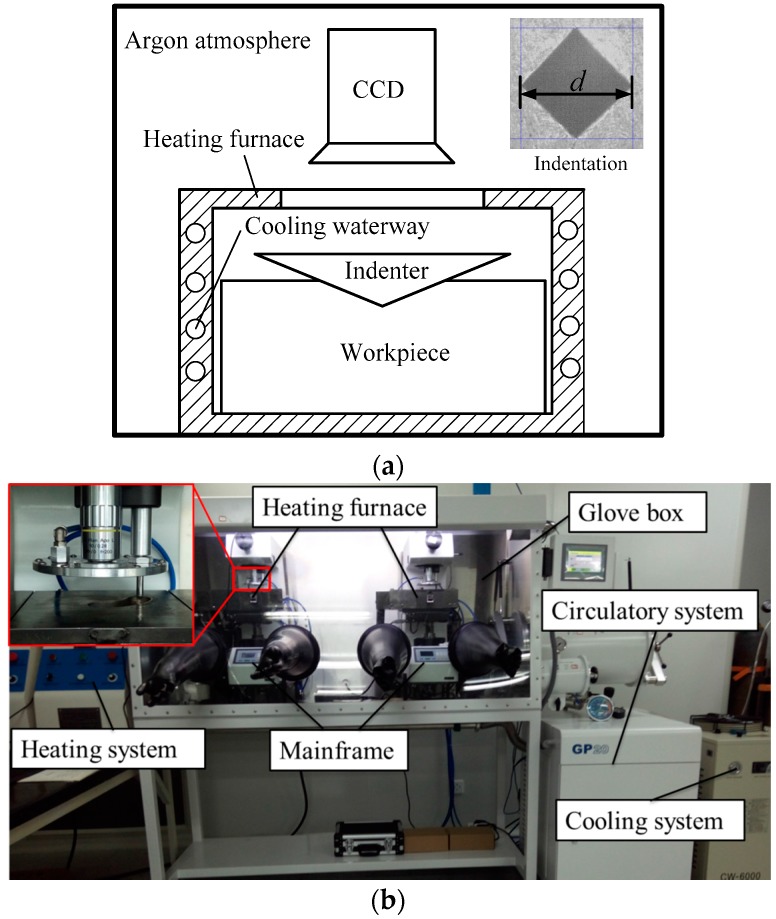
High-temperature hardness test principle (**a**) and equipment (**b**).

A series of high-temperature hardness experiments were carried out by a customed high-temperature hardness tester. The high-temperature hardness test principle and equipment are shown in Figure 3. The test temperature is provided by heating furnace with silicon molybdenum bar. The test temperature of this high-temperature hardness tester is up to 1000 °C, and the heating rate is up to 50 °C/min. In order to eliminate the effect of heat generated by the heating furnace on the mainframe of the high-temperature hardness tester, the cooling waterway is arranged outside the furnace chamber. The cooling waterway is connected to the cooling system to ensure that the cooling waterway has excellent cooling performance. The cooling system and the heating system work together to ensure that the temperature of the furnace chamber is maintained at a set value. In order to prevent the sample from being oxidized during the heating process, the mainframe of high-temperature hardness testers are placed in a glovebox, and the glovebox is filled with argon. Oxygen and water vapor will generate during the heating process, which could lead to the oxidation of the sample and the life reduction of the indenter. In order to solve this problem, the glovebox is connected to the circulatory system, which has the function of absorbing oxygen and moisture. The oxygen and water content are always less than 0.1 ppm during the experiment. In the test process, the sample is put into the heating furnace, then heated to the set temperature and insulation for 15 min. The indenter presses the sample under high temperature conditions and holds the load for 30 s, and an indentation is formed. The material of the indenter is nature diamond. The image acquisition system equipped with special light sources and a charge-coupled device (CCD) could acquire a length of indentation diagonal with the resolution of 1 μm under high temperature conditions. It is worth noting that the software of this high-temperature hardness test has the function of autofocus and automatic measurement, which guarantees measurement precision.

**Figure 4:**
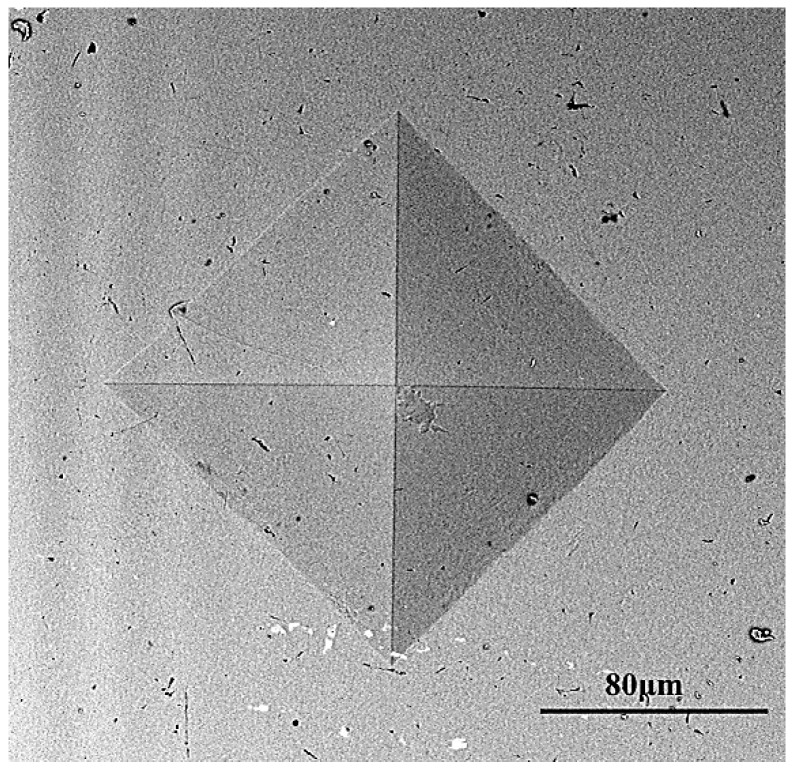
The topography of indentation under the test temperature of 800 °C (SEM).

High-temperature hardness tests of Fe-Cr-Ni stainless steel under different temperatures were performed. The design of the experiments is shown in Table 3. Each test is repeated three times to ensure the test precision. It is worth noting that there is a phase change around 700 °C for Fe-Cr-Ni stainless steel according to previous studies. The surface color of the material became dark, which leads to indentation size not being able to be measured by the high-temperature hardness tester. Thus, the test temperature of 700 °C is not listed in Table 3. The topography of indentation acquired by SEM under the test temperature of 800 °C is shown in Figure 4. It was found that the shape of the indentation was regular, that is, the the uniformity of the two diagonal line lengths of the indentation is better, which proved that the high-temperature hardness tester shows good performance and could provided accurate values of high-temperature hardness.

## 3. The Development of the FE Simulation Model

### 3.1. The Procedure of FE Simulation

**Figure 5:**
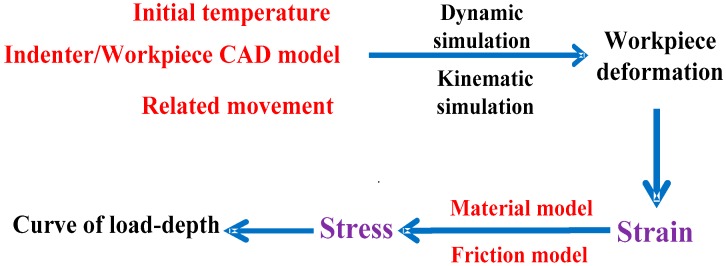
The procedure of numerical calculation.

High-temperature hardness simulations were performed with the commercial FEM software AdvantEdge^TM^ 7.1 (Third wave system, Wayne, NJ, USA), which integrates advanced finite element models for high-temperature hardness simulation. The software is developed by the third wave system company of United States. The procedure of numerical calculation is shown in Figure 5. The initial temperature, indenter/sample computer aided design (CAD)model and related movement between the indenter and sample were defined in the development process of the simulation model. The parameters of related movement include the initial related position, related movement speed and displacement. The CAD model of indenter and sample were developed by Pro/Engineer and then imported into AdvantEdge^TM^ with STEP format. The initial temperature and related movement could be defined in AdvantEdge^TM^ directly. The material model of the sample was developed based on the results of the quasi-compression experiments. At the same time, the friction model between the indenter and sample were developed and imported into AdvantEdge^TM^.

### 3.2. The Geometrical Model

**Figure 6:**
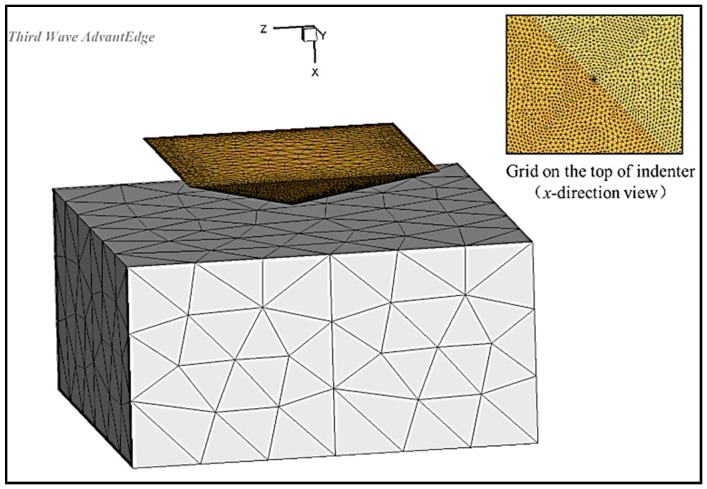
The geometrical model.

The CAD model of the workpice was imported into AdvantEdge^TM^, which is a cuboid with the dimensions of 1 mm × 1 mm × 0.6 mm. The CAD model of the indenter is a standard Vickers indenter with a tip angle of 136°. The initial related position is shown in Figure 6. The Vickers indenter is fixed in three directions, and the sample moved along the negative direction of the *x*-axis, while the *y*-axis and *z*-axis direction were fixed. The Lagrange finite element model based on adaptive meshing was used in the software AdvantEdge. The grid of sample and indenter were meshed into tetrahedrons. AdvantEdge^TM^ provides a convenient meshing process. Users only need to enter the maximum and minimum grid sizes and the parameter called mesh grading, which controls the transition speed from the maximum grid to the minimum grid, then the grid will be meshed automatically. At the same time, users can also customize the area of fine mesh. The grid at the tip of the indenter is meshed and the mesh is small enough to ensure the precision of the simulation, and the grid away from the tip is gradually larger, to improve the simulation efficiency. The adaptive finite element mesh is the most important feature of the AdvantEdge. The sample grid close to the grit is automatically refined appropriately and the grid away from the grit is refined slightly during the simulation. The simulation precision and efficiency could be guaranteed due to the features of AdvantEdge^TM^.

### 3.3. The Material Model

The Fe-Cr-Ni stainless steel and natural diamond were selected as the sample and indenter material, respectively. The material model of the Vickers indenter was offered by the material library of AdvantEdge^TM^. The material models of the sample were acquired by quasi-compression experiments.

The material constitutive model of the sample is one of the most important factors that affect the simulation precision. The material constitutive models used commonly include the Power law (P-L) model, Johnson–Cook model, Wright–Batra model et al. [30]. The constitutive model of sample material based on a P-L relationship which considers strain, strain rate and temperature was developed. The strain rate hardening could be ignored due to the fairly low strain rate in the process of the high-temperature hardness test, then the simplified material constitutive model based on the P-L relationship could be expressed as:

(1) $$\sigma_{s}=g(\varepsilon_{s})\times \Theta (T)$$

(2) g(εs)=σ0(1+εsε0)1n

(3) $$\Theta (T)=c_{0}+c_{1}T+c_{2}T^{2}+c_{3}T^{3}$$

where *σ_s_* is the flow stress; *g*(*ε_s_*) is strain strengthening items; Θ(*T*) is thermal softening items; *σ*_0_ is the yield stress at reference strain and reference temperature; *ε_s_* is the strain; *ε*_0_ is the reference strain; *n* is strain hardening factor, *T* is the temperature of sample. *c*_0_–*c*_3_ are the material constants of thermal softening items.

The true-stress–true-strain curve at room temperature is used to determine the material constant *σ*_0_ and *n*, in which the Equation (3) is assumed to be one. Then, Equation (1) could be simplified as:

(4) σs=σ0(1+εsε0)1n

*σ*_0_ is the yield strength, which is 1244.55 MPa determined directly from the true-stress–true-strain curve of 20 °C, shown in Figure 2. Two sides of Equation (4) are divided by *σ*_0_, then taking the logarithm of both sides, the equation could be as follows:

(5) $$\ln\frac{\sigma_{s}}{\sigma_{0}}=\frac{1}{n}\times \ln(1+\frac{\varepsilon_{s}}{\varepsilon_{0}})$$

**Figure 7:**
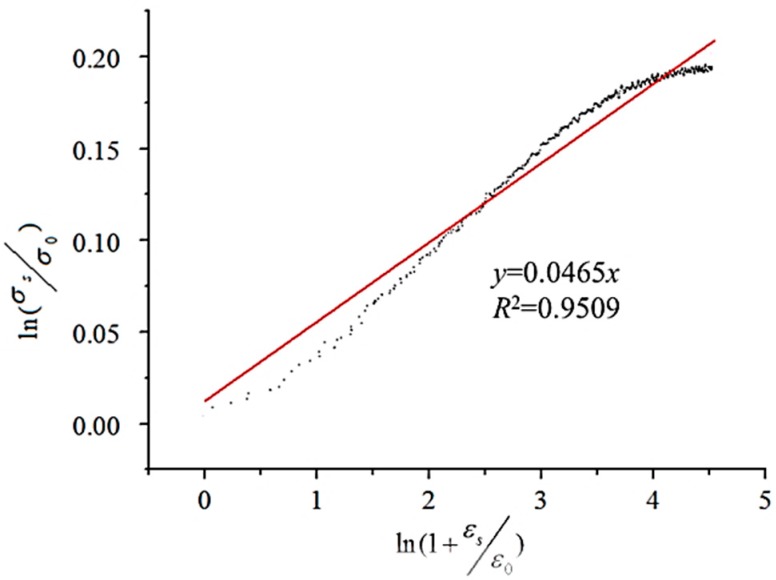
The relationship between ln(*σ_s_*/*σ*_0_) and ln(1 + *ε_s_*/*ε*_0_).

The relationship between ln(*σ_s_*/*σ*_0_) and ln(1 + *ε_s_*/*ε*_0_), and the plot of ln(*σ_s_*/*σ*_0_) − ln(1 + *ε_s_*/*ε*_0_) could be obtained when *ε*_0_ is determined, then the relationship between ln(*σ_s_*/*σ*_0_) and ln(1 + *ε_s_*/*ε*_0_) could be acquired by linear fitting. A lot of attempts have been made with different *ε*_0_. It was shown that when *ε*_0_ is 0.003, *R*^2^ has a maximum value of 0.9509, which is shown in Figure 7. The value of 1/*n* is the slope of the line which is fitted. Then, the material constant *n* is determined as 21.510.

**Figure 8:**
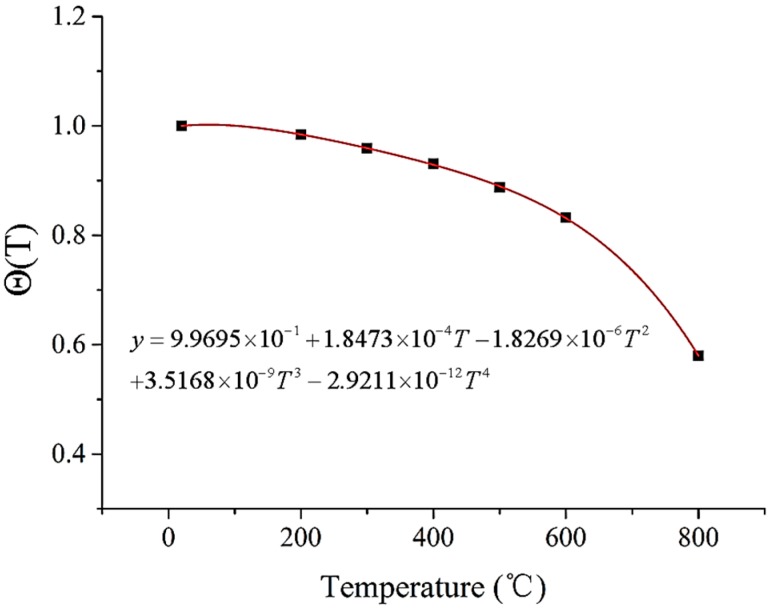
The fitting result of thermal softening.

It was found that the true stress decreases with the increase of true strain when the true strain is greater than 0.3 in, see Figure 1. The reason for this is that the samples are damaged when the true strain is greater than 0.3. In order to more fully reflect the thermal softening of the sample material, the values of true stress when the true strain is 0.2 under different temperatures are used to fit Equation (3) by the polynomial fitting method. The result of the fitting is shown in Figure 8.

Thus, the material constitutive model of the sample could be expressed as follows:

(6) σs=1244.55(1+εs0.003)121.510×(9.9695e−1+1.8473e−4T−1.8269e−6T2+3.5168e−9T3−2.9211e−12T4)

### 3.4. The Friction Model

**Figure 9:**
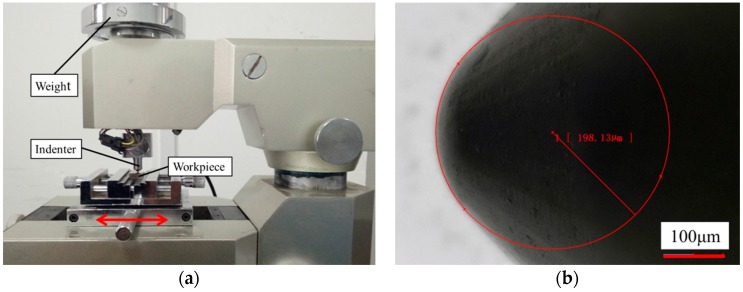
The set-up and indenter for the measurement of the friction coefficient.

The friction coefficient between indenter and sample has an effect on the indentation hardness test [31]. The friction coefficient measurement between the Vickers indenter and sample was performed on a scratch tester, as shown in Figure 9a. The reciprocal friction mode was selected to measure the friction coefficient. The Rockwell indenter, which is made of nature diamond, was selected as a frictional pair. The tip radius of the Rockwell indenter was 0.2 mm, as shown in Figure 9b. The other frictional pairs was cuboid with dimensions of 16 mm × 16 mm × 8 mm, and made of Fe-Cr-Ni stainless steel. The normal load is provided by the weight, and the tester could record the friction force in real time, to calculate the friction coefficient. The friction coefficient is related to the test load and relative velocity. The relative velocity between the workpiece material and Vickers indenter is extremely slow during the hardness test, which could be considered a quasi-static process. The speed of the reciprocating movement was set as low as possible during the measurement of the friction coefficient. A series of experiments were carried out to research the effect of load on the friction coefficient and the design of experiment is shown in Table 4.

**Figure 10:**
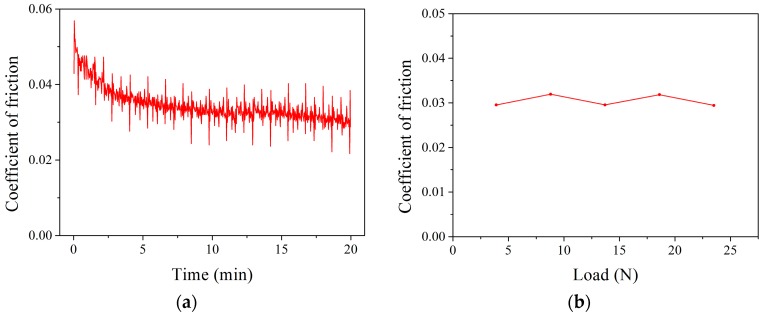
The curve of friction coefficient (**a**) and the relationship between the friction coefficient and load (**b**) All the simulation models needed to have been developed so that the high-temperature hardness simulation could be carried out. High-temperature hardness simulations at different temperatures were performed by importing different initial temperatures, and the design of the simulation is shown in Table 5.

The measurement results are shown in Figure 10. It was found that the friction coefficient decreases with the increase of friction time, and then the friction coefficient is gradually stable. It was also found that the friction coefficient almost does not change with the change of load. The friction coefficient is determined to be 0.03, according to the results shown in Figure 10b.

**Figure 11:**
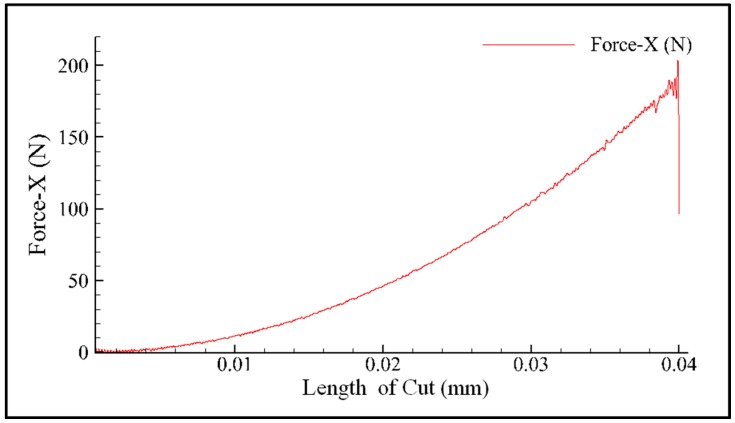
The result of the AdvantEdge^TM^ outputted (initial temperature = 20 °C).

The force applied at the indenter-pressing depth curve could be outputted directly, as shown in Figure 11. According to the geometric shape of the standard Vickers indenter, the relationship between the pressing depth of indenter and the diagonal length of the indentation can be expressed as:

(7) d=7.0006h

where *d* is the diagonal length of the indentation and *h* is the pressing depth of the indenter. The relationship of the value of Vickers hardness and the diagonal length of the indentation is expressed as follows:

(8) $$HV=0.1891\frac{F}{d^{2}}$$

where *F* is the force applied to the indenter.

According to the above analysis, a conclusion could be drawn that a high-temperature hardness numerical simulation could provide the high-temperature hardness of all loads under simulated temperature.

## 4. Results and Discussion

**Figure 12:**
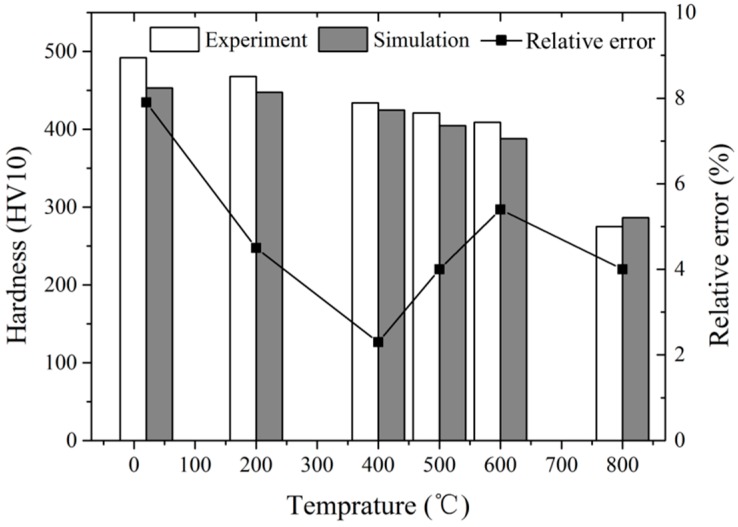
Comparison of simulation and experiment results.

The results of the high-temperature hardness experiment and simulation under the load of 10 kg were compared to verify the accuracy of the high temperature indentation FE simulation, and the comparison result is shown in Figure 12. The average relative error of different temperatures is 4.68%, and the maximum relative error is less than 8%.

**Figure 13:**
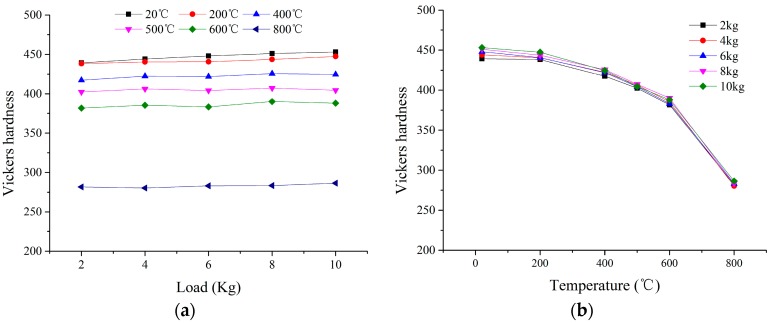
The Vickers hardness under different loads (**a**) and temperatures (**b**).

The high-temperature hardness under different loads and temperatures acquired by simulation is shown is Figure 13. It was found that the Vickers hardness under different temperatures increases slightly with the increase in the load, and the increase is extremely small. The material pile-up degree at the edge of the indentation increases with the increase in load, and the additional contact area of the indenter with the material due to the pile-up increases with the increase in the load, which leads to the slight increase. The conclusion could be drawn that the high temperature hardness basically does not change with the change of load when the pile-up is ignored. It was also found that the Vickers hardness under different loads decreases with the increase of temperature, and the decrease rate increases with the increase of temperature. At the same time, the variation of Vickers hardness under different loads with the change of temperature is consistent.

**Figure 14:**
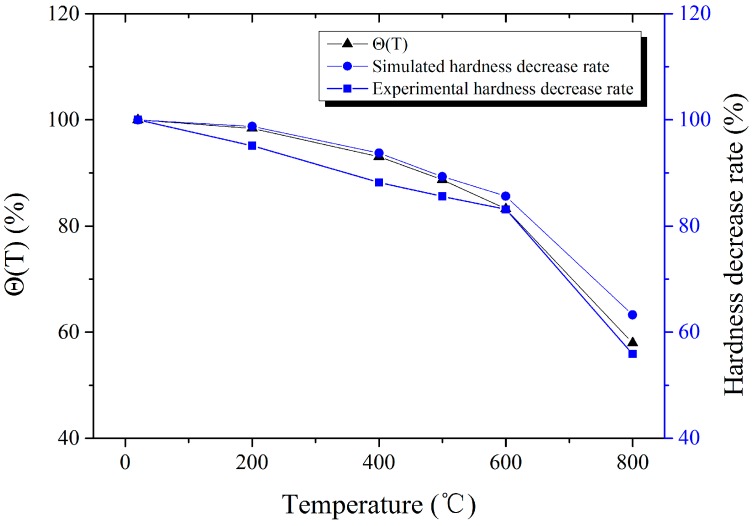
The comparison of experimental hardness decrease rate and Θ(*T*).

The ratio of Vickers hardness under high temperature and that under room temperature (20 °C) is called the hardness decrease rate. Θ(*T*) is the thermal softening of items acquired by the high-temperature, quasi-static compression experiment described in Section 2.1. The comparison of the experimental hardness decrease rate, simulated hardness decrease rate (load = 10 kg) and Θ(*T*) is shown in Figure 14. It was found that the the hardness decrease rate corresponds well with Θ(*T*), and the slight difference is mainly due to the experimental error of high-temperature hardness and high-temperature, quasi-static compression. The conclusion could be drawn that the hardness decrease rate and thermal softening are consistent.

**Figure 15:**
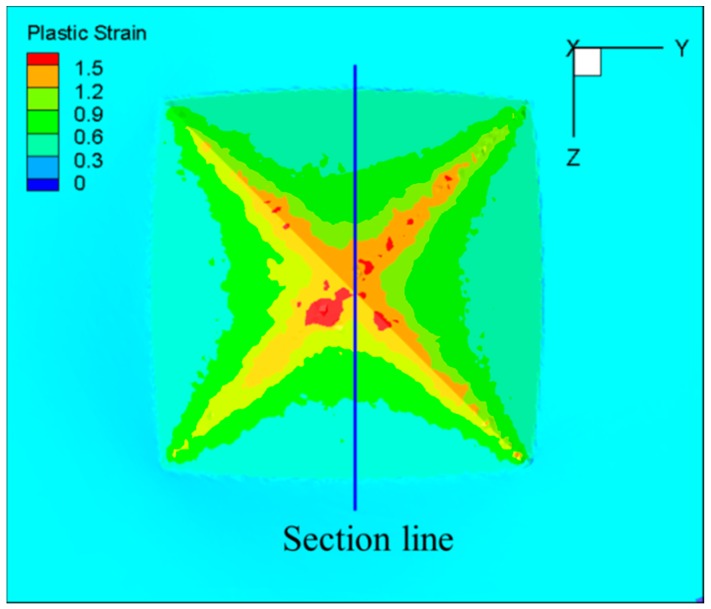
Strain contour of indentation (Temperature = 20 °C, Load = 10 kg).

**Figure 16:**
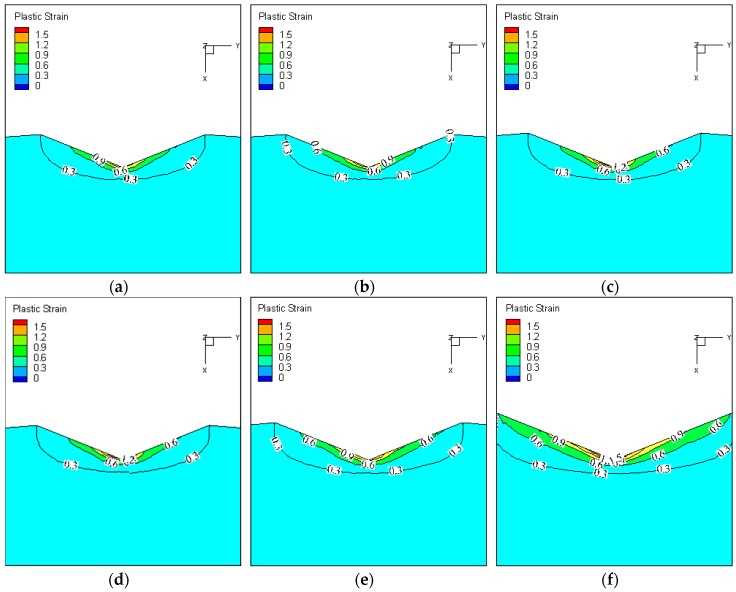
Strain contour of indentation section under different test temperatures (Load = 10 kg). (**a**) Temperature = 20 °C; (**b**) Temperature = 200 °C; (**c**) Temperature = 400 °C; (**d**) Temperature = 500 °C; (**e**) Temperature = 600 °C; (**f**) Temperature = 800 °C.

FE simulation provides a means of acquiring some physical quantities which can not be measured by experiment, such as stress and strain. The strain distribution of the indentation is shown in Figure 15. It was found that the material deformation is not uniform in the Vickers hardness test process. The deformation of the material which contacts with the edges of the indenter is more severe, and the strain near the top of indenter is larger than that of the other parts. In order to obtain the strain distribution inside the indentation, the indentations were cut along the section line marked in Figure 15. The strain contour of the indentation section under different test temperatures (Load = 10 kg) is shown in Figure 16. It was found that the form of strain distribution of the indentation under different temperatures was the same, and the strain increases with the increase of test temperature. The material is softened at high temperature, and the depth of indentation increases with the increase of test temperature, which leads to the increase in strain.

**Figure 17:**
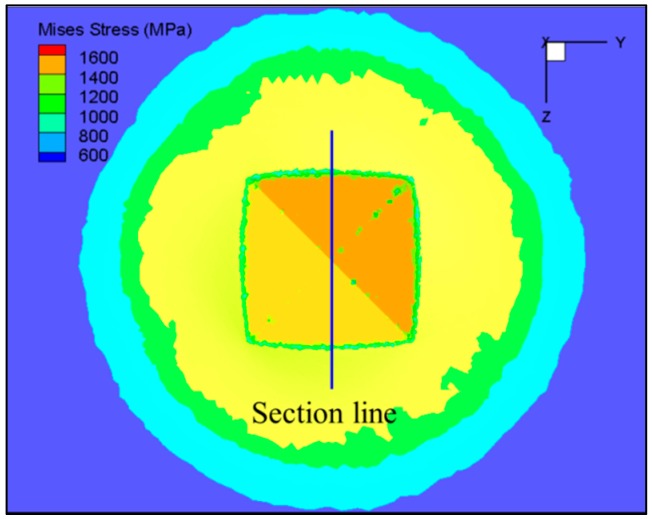
Stress contour of indentation (Temperature = 20 °C, Load = 10 kg).

**Figure 18:**
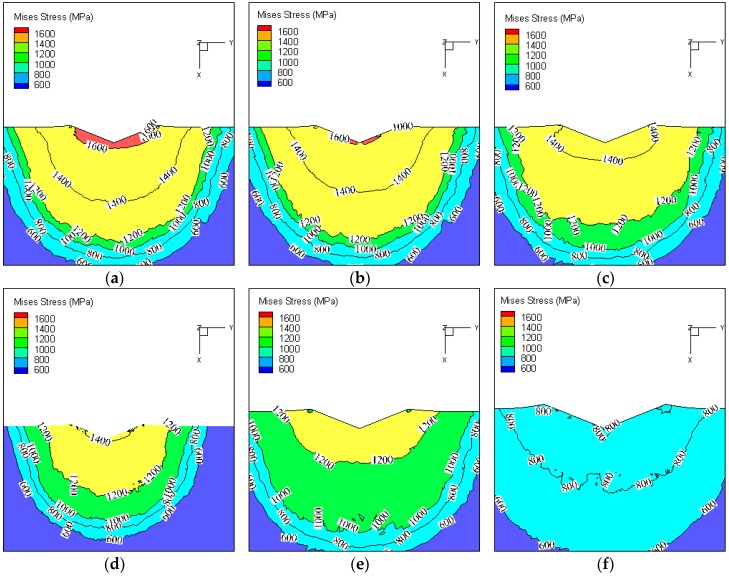
Stress contour of indentation section under different test temperatures (Load = 10 kg). (**a**) Temperature = 20 °C; (**b**) Temperature = 200 °C; (**c**) Temperature = 400 °C; (**d**) Temperature = 500 °C; (**e**) Temperature = 600 °C; (**f**) Temperature = 800 °C.

The stress distribution of the indentation is shown in Figure 17. In order to obtain the stress distribution inside the indentation, the indentations are cut along the section line marked in Figure 17. The stress contour of the indentation section under different test temperatures (Load = 10 kg) is shown in Figure 18. It was found that the stress decreases with the increase of test temperature. The material is softened at high temperature, and the depth of indentation and the contact area between the indenter and the sample increase with the increase of test temperature. Thus, the stress decreases with the increase of test temperature under the same load. 

## 5. Conclusions

- An FEM model of the relationship between high-temperature hardness and high-temperature, quasi-static compression experiment was developed.
- The simulated and experimental results of high temperature hardness were compared, which verified the accuracy of the high-temperature indentation FE simulation.
- The high-temperature hardness basically does not change with the change of load when the pile-up is ignored, and the hardness decrease rate and thermal softening are consistent.

## Figures and Tables

**Table 1:** The chemical composition of the sample material.

Composition	C	Si	Mn	P	S	Ni	Cr	Mo	V	N	Nb	Fe
Content	0.17	<0.07	<0.30	<0.01	<0.01	3.5	11.50	3.00	0.28	0.07	0.12	Balance

**Table 2:** Design of experiment for uniaxial compression experiments.

Items	Parameters
Sample size (mm)	Diameter: 5, Height: 5 (cylindrical specimen)
Compression speed (mm/min)	0.3
Insulation time (min)	15
Test temperature (°C)	20, 200, 300, 400, 500, 600, 800

**Table 3:** Design of experiments for high-temperature hardness tests.

Items	Parameters
Test load (kg)	10
The time to keep the temperature (min)	15
The time to keep the load (s)	30
Test temperature (°C)	20, 200, 400, 500, 600, 800

**Table 4:** Design of the experiment for the measurement of the friction coefficient.

Items	Parameters
Reciprocating stroke (mm)	6
Reciprocating speed (mm/s)	0.5
Load (N)	4.9, 9.8, 14.7,19.6, 24.5

**Table 5:** Design of simulation for high-temperature hardness.

Items	Parameters
Pressing depth (μm)	40
Pressing speed (mm/s)	0.3
Initial temperature	20, 200, 400, 500, 600, 800
